# Genome-Wide DNA Methylation Profiling in the Lotus (*Nelumbo nucifera*) Flower Showing its Contribution to the Stamen Petaloid

**DOI:** 10.3390/plants8050135

**Published:** 2019-05-20

**Authors:** Zhongyuan Lin, Meihui Liu, Rebecca Njeri Damaris, Tonny Maraga Nyong’a, Dingding Cao, Kefang Ou, Pingfang Yang

**Affiliations:** 1 Key Laboratory of Plant Germplasm Enhancement and Specialty Agriculture, Wuhan Botanical Garden, Chinese Academy of Sciences, Wuhan 430074, China; lzygry@sina.com (Z.L.); 18292011525@163.com (M.L.); njerirebecca09@gmail.com (R.N.D.); maragatonny@gmail.com (T.M.N.); caodingding@wbgcas.cn (D.C.); 2 University of Chinese Academy of Sciences, Beijing 100039, China; 3 Wuhan Institute of Landscape Architecture, Wuhan 430081, China; Oukefang@163.com; 4 State Key Laboratory of Biocatalysis and Enzyme Engineering, School of Life Sciences, Hubei University, Wuhan 430062, China

**Keywords:** DNA methylation, *Nelumbo nucifera*, whole genome bisulfite sequencing, gene expression

## Abstract

DNA methylation is a vital epigenetic modification. Methylation has a significant effect on the gene expression influencing the regulation of different physiological processes. Current studies on DNA methylation have been conducted on model plants. Lotus (*Nelumbo nucifera*) is a basic eudicot exhibiting variations during development, especially in flower formation. DNA methylation profiling was conducted on different flower tissues of lotuses through whole genome bisulfite sequencing (WGBS) to investigate the effects of DNA methylation on its stamen petaloid. A map of methylated cytosines at the single base pair resolution for the lotus was constructed. When the stamen was compared with the stamen petaloid, the DNA methylation exhibited a global decrease. Genome-wide relationship analysis between DNA methylation and gene expression identified 31 different methylation region (DMR)-associated genes, which might play crucial roles in floral organ formation, especially in the stamen petaloid. One out of 31 DMR-associated genes, NNU_05638 was homolog with Plant U-box 33 (PUB33). The DNA methylation status of NNU_05638 promoter was distinct in three floral organs, which was confirmed by traditional bisulfite sequencing. These results provide further insights about the regulation of stamen petaloids at the epigenetic level in lotus.

## 1. Introduction

DNA methylation is one of the vital features of epigenetic modification, which plays a key role in plant growth and development through its regulation of gene expression [1,2,3]. DNA methylation occurs in all cytosine sequence contexts including CG and non-CG (CHG and CHH, where H represents any nucleotide but G) in plant genomes [4]. These three sequence contexts are maintained by individual enzymatic pathways [5]. CG methylation is maintained by methyltransferase 1 (MET1), the plant homolog of DNA methyltransferase 1 (DNMT1) in animals [6]. Non-CG methylation is abundant in plants compared to mammals and is site specific. DNA methylation in CHG and CHH is mediated by CHROMOMETHYLASE (CMT) and DOMAINS REARRANGED METHYLTRANSFERASE (DRM) proteins, respectively [7]. CMT3 is controlled by histone H3 lysine 9 (H3K9) methylation [4,6]. DRM2 is targeted to a certain site through short-interfering RNAs (siRNAs) produced by the RNA-directed DNA methylation (RdDM) pathway in the plant [8,9]. 

Numerous methods have been developed to examine the DNA methylation level, among which bisulfite sequencing is regarded as an excellent method [10]. Previous studies have reported whole genome bisulfite sequencing (WGBS) and the methylation profile in many plants, including *Arabidopsis*, rice, soybean, maize, and mung beans [11,12,13,14,15]. DNA methylation exhibits dynamic features with tissue and developmental specificity, which has a fundamental role in the transcriptional control of genes and repeats [16]. However, for the lotus (*Nelumbo nucifera*), a basal taxon of the extant eudicot plant, the DNA methylation patterns and functions in different biological processes have not been studied. Additionally, the relationship between DNA methylation and gene expression remains obscure in the lotus.

Because of its importance in phylogenetics, the lotus genome has been sequenced and annotated, providing a basis for further studies on this species. Previous studies have reported roles of various types of transcriptomes and proteomes in regard to the flower coloration mechanism [17], seed development [18], and rhizome development [19] of lotus. Deng et al. reported the existence of different DNA methylation intensities on the promoter region of the *ANS* gene in different flower colorations of the red and white cultivar [17]. However, no studies have explored the effects of DNA methylation on gene expression. Therefore, this study examined the DNA methylome of different lotus flower tissues by deep bisulfite sequencing and compared the distribution and average level of DNA methylation in CG, CHG, and CHH contexts. Our analysis focused on the whole genome DNA methylation pattern and performed genome-wide relationship analysis between DNA methylation and gene expression. Several critical genes that might be involved in the regulation of flower organ development were determined based on the methylation patterns. According to our results, we propose that effects of DNA methylation occur via regulation of gene expression to control the petaloid formation. These results will deepen the understanding of DNA methylation influence on gene expression.

## 2. Results and Discussion

**Figure 1:**
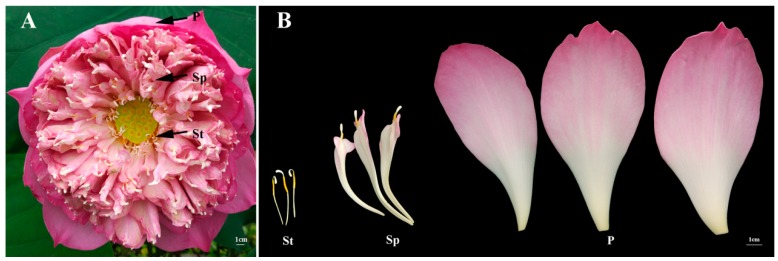
Flower of ‘Fenhonglingxiao’. (**A**) The blossom flower of ‘Fenhonglingxiao’. (**B**) Three floral organs of ‘Fenhonglingxiao’. P represents petal; Sp represents stamen petaloid; St represents stamen. Bars are all 1 cm.

Lotus (*Nelumbo nucifera*) flowers are brightly colorful, with several of them exhibiting special morphologies and flower color or form. Particularly, the phenomenon of the petaloid stamen is common. For example, a lotus flower cultivar ‘Fenhonglingxiao’ belongs to the tropical lotus, which has big sizes of flowers and possesses many petaloid stamens (Figure 1). Developmental anomalies in the plant are reported to be influenced by changes in DNA methylation [20]. DNA methylation levels and landscape mostly depend on DNA methyltransferase and DNA demethylase.

### 2.1. Expression Levels of Multiple Enzymes Involvement in DNA Methylation

**Figure 2:**
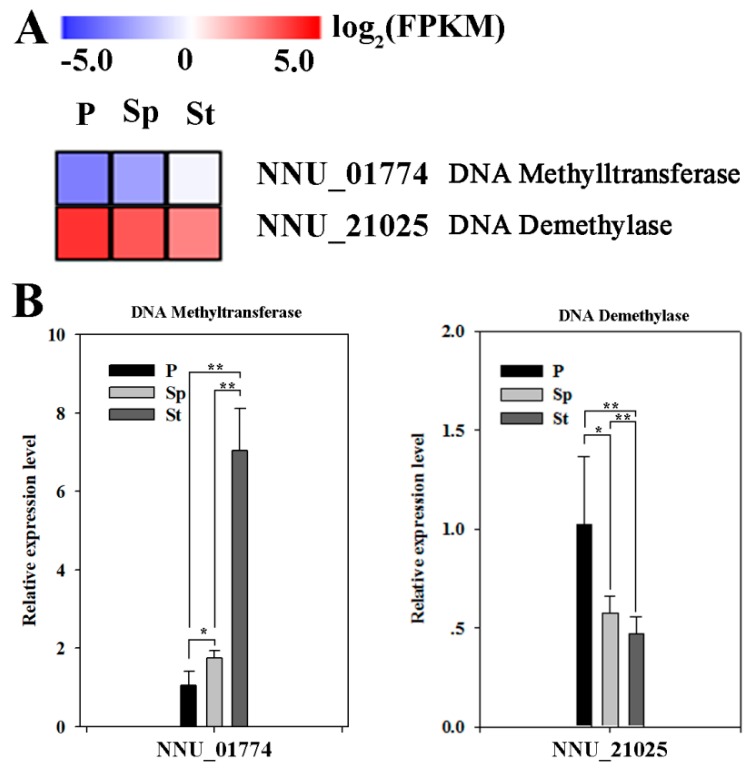
The expression level of DNA methyltransferase and DNA demethylase genes. (**A**) The heatmap of DNA methyltransferase and DNA demethylase genes was constructed by the FPKM value. (**B**) The relative expressions of DNA methyltransferase and DNA demethylase genes were detected by qRT-PCR in three floral organs. Relative gene expressions were normalized by comparison with the expression of lotus β-actin (NNU_24864) and using the 2^-ΔΔCT^ method. The error bars represent the SD for three biological replicates. Asterisks indicate the statistical significance of the indicated differences (* *p* < 0.05; ** *p* < 0.001; T test).

The RNA-seq data of the petal (P), stamen petaloid (Sp), and stamen (St), was previously reported by Lin et al. [21]. From this data, one DNA methyltransferase (NNU_01774) and one DNA demethylase (NNU_21025) were found in differential expression genes (DEGs). Fragments per kilo base of transcript per million base pairs sequenced (FPKM) value from RNA-seq data and quantitative real time PCR (qRT-PCR) data both show that the expression of one DNA methyltransferase gene gradually increased in P, Sp, and St with one DNA demethylase gene expression showing an opposite pattern (Figure 2). These results demonstrate that DNA methylation may be implicated in petaloid formation. 

### 2.2. Whole Methylome Sequencing and Genome Methylation Profiles of the Lotus

To verify the lotus methylomes, whole genome bisulfite sequencing was performed using Illumina sequencing on the genomic DNA isolated from the P, Sp, and St of fully opened flower from the flower lotus cultivar, ‘Fenhonglingxiao’ (Figure 1), which was generated by a DNA methylation map across the lotus genome. A total of 122.17 million, 119.69 million, and 117.77 million raw reads were obtained from P, Sp, and St, respectively (Table S1). Raw reads were filtered through Trimmomatic software as clean data ensuring sequence accuracy. The clean reads were mapped to the ‘China Antique’ lotus reference genome using Bismark software [22,23]. Thus, the final mapped reads were ~88.73 million, ~86.89 million, and ~86.79 million for P, Sp and St, respectively (Table S1), with more than 74% aligned to the lotus reference genome. 

**Figure 3:**
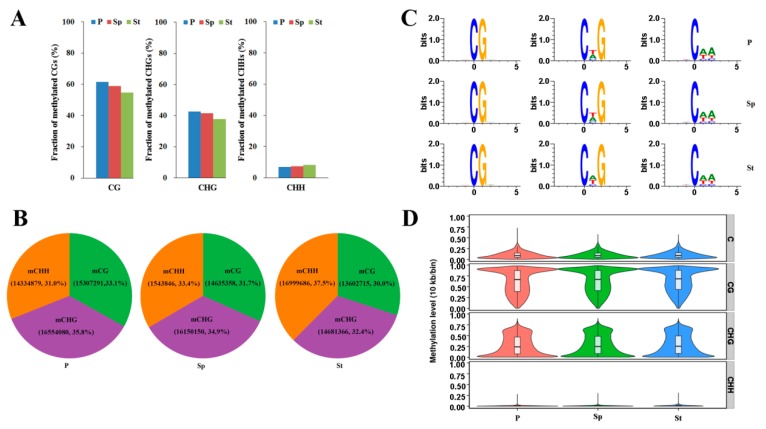
DNA Methylation patterns in the floral organs of the lotus. (**A**) The fraction of methylated cytosines in different contexts. (**B**) The percentage of methylcytosines identified in different sequence contexts for Petal (P), Stamen petaloid (Sp), and Stamen (St). (**C**) DNA sequence logo plot of the methylated cytosine contexts. Information was for the 9 bp base around the position of the methylated cytosine contexts. (**D**) The methylation level violin in each sequence context for P, Sp, and St. The x axis represents different samples. The y axis represents methylation level. 10 kb was considered as one bin. The width of each violin represents the number of C sites at this methylation level.

There was a slight difference in methylated cytosines in all the contexts in the genome between any two tissues (Figure 3a). The ratio of cytosine methylation was ~58.4%, ~40.6%, and ~7.5% for the CG, CHG, and CHH contexts, respectively (Figure 3a). The sacred lotus methylome contains different proportions of methylated methylcytosines (mCs) in the petal (33.14% mCG, 35.83% mCHG, 31.03% mCHH), stamen petaloid (31.66% mCG, 34.94% mCHG, 33.39% mCHH), and stamen (30.04% mCG, 32.42% mCHG, 37.54% mCHH) (Figure 3b and Table S2). In the lotus, the proportion of methylated cytosines was fairly equal and more similar to soybean than the other plants (Figure S1). Notably, the proportion of mCG sites in the lotus is higher compared to the brich and mung bean, but lower than soybean and *Arabidopsis*, which have the mCG sites as the predominant methylation sites [11,15,24,25] (Figure S1). For analysis of the relationship between sequence context and methylation preference in the lotus, we calculated the percentage of methylation for all possible 9 bps in which methylated cytosine was in the fifth position (allowing an analysis of four nucleotides upstream of CG, CHG, and CHH methylation). In all mCGs, there were common sequences in the three floral organs, with an exception for the CHG context in St (Figure 3c). This result indicated that the petaloid stamen has more similar methylation preference with the petal than with the stamen, suggesting that DNA methylation may be associated with petaloid formation. However, in the CHH context with high methylation, the petaloid stamen still maintained the methylation preference equal to the stamen, which was consistent with the petaloid stamen that also possessed the stamen feature (Figure 1 and Figure S2). The results indicate that different methylation patterns are tissue specific.

### 2.3. Methylation Profile of Genes

The weighted methylation levels were calculated to reveal the distribution of DNA methylation around genes. The methylation level violin of whole genes in the lotus genome showed different patterns depending on the cytosine contexts, but similar patterns in different tissues (Figure 3d). The mCG and mCHG displayed an opposite violin state, and mCHH showed a narrow spectrum of methylation levels in each width of the violin.

**Figure 4:**
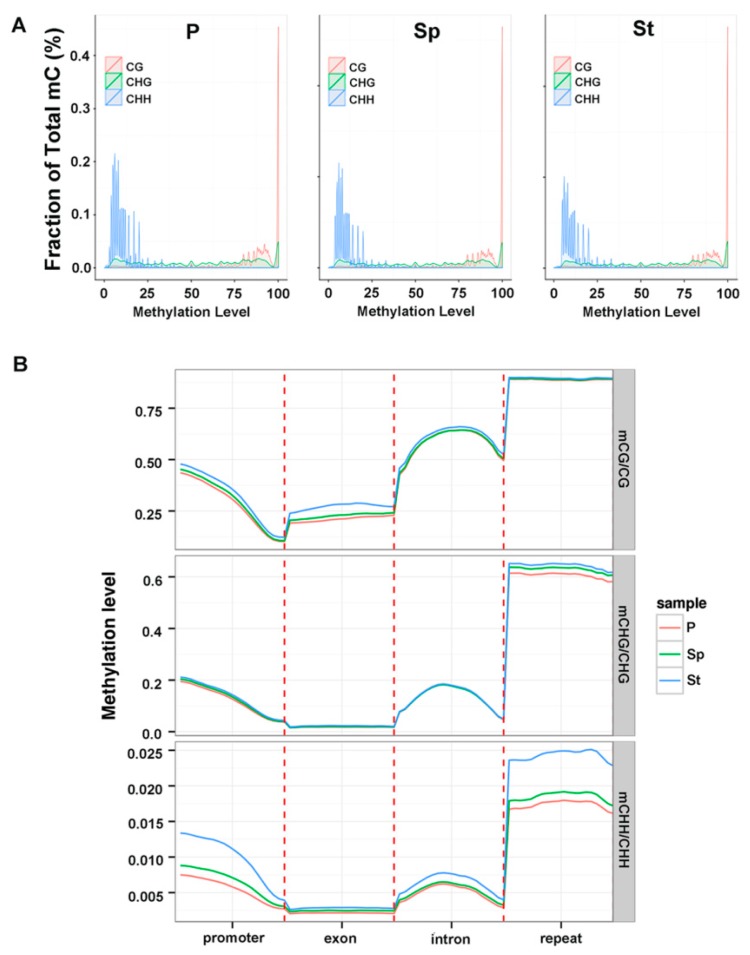
The Genomic feature of methylation level in lotus tissues. (**A**) Distribution of methylation level of mCs in each sequence context. (**B**) Methylation level of different genomic regions (promoter, exon, intron and repeat) in each cytosine context. The promoter region is an upstream 2 kb sequence from transcription starting site (TSS).

In lotus, there is a tendency of highly methylated CG sites ranging between 75–100%, with CHG sites showing a more uniform distribution in different methylation level, and CHH having a low methylation of 0–25% (Figure 4a). They have the same distribution pattern in distinct methylation levels under each cytosine context in three floral organs (Figure 4a). In general, mCG and mCHG are methylated at higher levels as compared to mCHH. The phenomenon was similar to the methylcytosines in *Arabidopsis* [25]. The distribution of methylated cytosine in all contexts showed similar patterns in different organs in the main chromosome (Figure S3). 

We analyzed the methylation level of different genomic regions (promoter, exon, intron, and repeat) in mCG, mCHG, and mCHH contexts and found that the exon regions generally have less methylation (Figure 4b). In mCG and mCHG, the methylation levels in different genomic regions had a similar pattern among the three floral organs, with the exception of mCG on the exon regions containing a slightly higher level of methylation in St. Specifically in the CHH context, there was a clear difference in the methylation level in the promoter, intron, and the repeat regions among the three samples, with St containing the highest level of methylation. Impressively, the methylation level in introns was higher than those in exons, which was opposite to *A. thaliana* [26] but similar to brich [24]. This high enrichment in the intron indicated that DNA methylation could have a complex regulation in the lotus. Methylation in the different genomic regions of P and Sp exhibited high levels of resemblance while low or no resemblance was observed in St (Figure S4). Some DNA methylation regions on the whole genome had different levels in the three organs. The above states revealed that DNA methylation often occurred in the CG context and in a non-CG context throughout all the chromosomes or the functional genome region of the lotus (Figure 3 and Figure 4) consistent with the DNA methylation level depicted in other plants.

### 2.4. DEG Expression Associated with DNA Methylation

**Figure 5:**
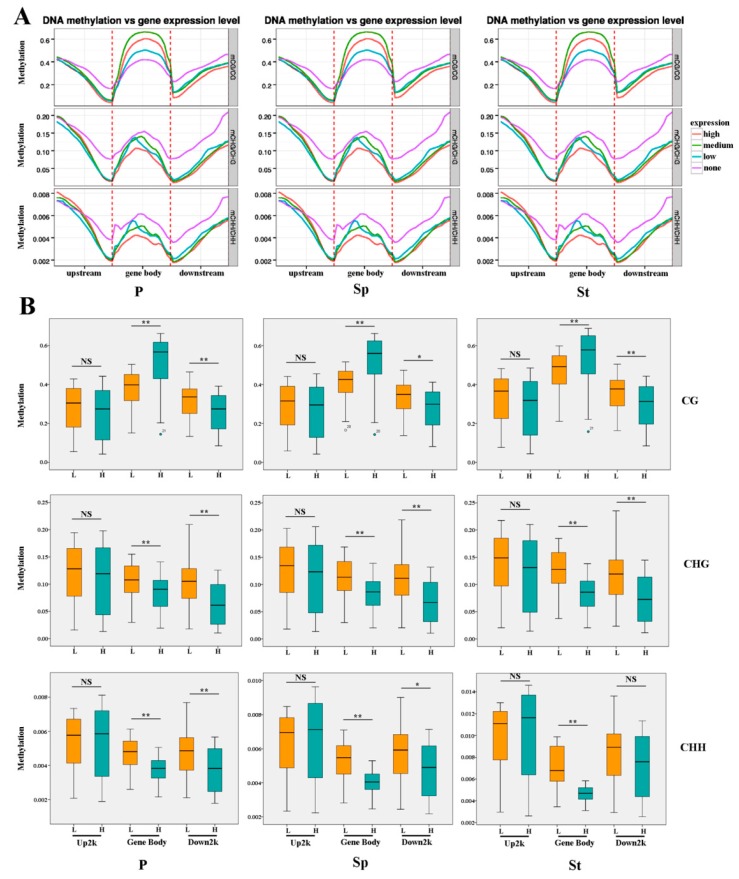
Methylation at various genic regions differentially associated with gene expression. (**A**) Comparison of gene expression and methylation levels for mCG, mCHG, mCHH sites and for each genic region: upstream 2 kb regions (Up2k), gene body, and downstream 2 kb regions (Down2K). On the basis of the expression of the upper and downer quantile, the genes were divided into four group: none (FPKM < 1); low (1 < FPKM < downer quantile); medium (downer quantile < FPKM < upper quantile); high (FPKM > upper quantile). The default selection FPKM = 1 as threshold of gene is expressed. (**B**) The box plot of gene expression and methylation levels for mCG, mCHG, mCHH sites and for each genic region: upstream 2 kb regions (Up), gene body, and downstream 2 kb regions (Down). Low expression (L) included none (FPKM < 1) and low (1 < FPKM < downer quantile); High expression (H) included medium (downer quantile < FPKM < upper quantile) and high (FPKM > upper quantile). Asterisks indicate the statistical significance of the indicated differences (NS: not significant; *: *p* < 0.05; **: *p* < 0.001; Mann-Whitney U test).

Several studies have shown that DNA methylation in different genomic regions is distinct and correlate with gene expression [1,27,28]. To confirm the relationship between gene expression and DNA methylation, DNA methylation was measured through WGBS using the same samples used for gene expression profiling. A DNA methylation profile throughout the gene region was examined. For upstream 2 kb and downstream 2 kb flanking sequences, the DNA methylation levels dramatically increased, departing from the transcription starting sites (TSSs) and termination sites in all contexts (Figure 5a). Regions between gene bodies and up- or downstream of promoters displayed a dramatic decline in methylation level. Among the three types of methylation, mCG was the highest, while mCHH was the lowest (Figure 5). In addition, CG methylation also showed a higher level in the gene body (Figure 5a). The DNA methylation level was calculated in the upstream (2 kb), gene body, and downstream (2 kb) regions, and the gene expression abundance was plotted against the DNA methylation level in all contexts (Figure 5a,b). According to the gene expression, the DEGs were grouped into four levels (Figure 5a). Genes with no expression had the highest methylation in the regions containing TSSs and termination sites, while genes with medium expression showed the highest CG methylation level on the gene body. These results are consistent with previous studies in rice and *Arabidopsis* [26,29]. In CG methylation, the methylation levels and gene expression had a negative correlation in the upstream and downstream sequence and a positive correlation in the gene body (Figure 5b). In contrast to CG methylation, the CHG methylation level was negatively correlated with gene expression in the whole gene region (Figure 5b). In CHH methylation, there were opposite patterns in the upstream and gene bodies with CG methylation, but they had similar profiles in the downstream region (Figure 5b). The methylation of the upstream 2 kb region was not significant in the different context sequences of different floral organs (Figure 5b). Contrary to the previous speculations [30,31], these results demonstrate the existence of an intricate relationship between DNA methylation and gene expression. Methylation levels at CG, CHG, and CHH contexts were almost indistinguishable in different floral organs (Figure 5a,b). Promoter region methylation is regularly associated with gene expression inhibition, while the function of the gene body methylation remains elusive, though both positive and negative relationships have been reported [1,29]. However, methylation in rice revealed that gene body methylation was positively associated with gene expression [32]. Meng et al. reported that DNA methylation is slightly conducive to gene expression [33]. A recent study conducted on apples did not uncover a correlation between promoter methylation and gene expression, whereas it showed a positive relationship between the gene body methylation and gene expression [34]. Our observation shows that the incompatible incidences could be a result of the interaction of DNA methylation with other factors [6,7,13]. The CG and CHG contexts have similar DNA methylation patterns in three floral organs, except in the gene body of the CG context. When compared to P and Sp, the composition of DEGs in the St DNA density was prominent (Figure S5a). Moreover, for DNA methylation, St had the highest levels compared to the other two organs while the lowest level was evident in P, which was similar to a previous study exhibiting more robust maintenance of CG methylation in pollen [35]. Unlike CG and CHG methylation, the methylation in CHH was significantly different among P, Sp, and St, regardless of the DNA methylation density or level (Figure S5a,b), suggesting that CHH methylation might be crucial for floral organ gene expression. This correlates with the previous study on *Arabidopsis* [1]. The results showed that Sp had a similar DNA methylation profile with P compared to St. We observed that methylation profiles in the whole genome were similar in different floral organs of the lotus. A recent review summarized that the difference in methylation levels was minor in all context in several similar type organs, like vegetative organs [36]. However, the observations that methylation patterns and expression changes in methylation differ depending on the sequence contexts and organs, suggesting complex relationships between methylation and organ-specific gene functions.

### 2.5. Distribution of DNA Methylation Variation

**Figure 6:**
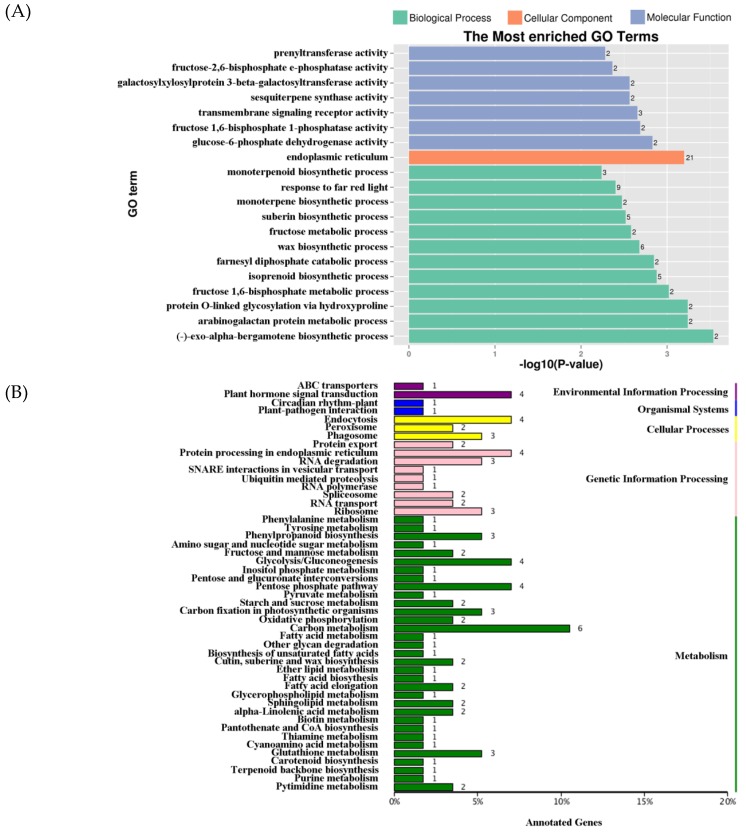
The most enriched Gene Ontology (GO) terms (**A**) and Kyoto Encyclopedia of Genes and Genomes (KEGG) classification (**B**) in all DMR-related genes.

To identify differential methylation regions (DMRs) among organs, we calculated the fractional methylation level of 1000 bp window with a 100 bp step length. We also identified 351 DMR-associated genes, included 48, 49, and 318 in P vs Sp, Sp vs St, and P vs St, respectively. The Gene Ontology (GO) enrichment analysis was conducted for total DMR-associated genes, which was mapped to the reference genome (Figure 6a). The results revealed genes mainly involved in endoplasmic reticulum (Figure 6a). These results suggest that DNA methylation may influence cellular components in different floral organs. The Kyoto Encyclopedia of Genes and Genomes (KEGG) classification was also performed (Figure 6b). DMR-associated genes were majorly classified to ‘Plant hormone signal transduction’, ‘Endocytosis’, ‘Protein processing in endoplasmic reticulum’, ‘Glycolysis/Gluconeogenesis’, ‘Pentose phosphate pathway’, and ‘Carbon metabolism’ (Figure 6b). In plant hormone signal transduction, there were four DMR-associated genes including NNU_00312, NNU_02663, NNU_04718, and NNU_05583. Moreover, NNU_02663, NNU_04718, and NNU_05583 were shown to be involved in the auxin pathway, which is critical to cell enlargement and plant growth. These findings demonstrate that they may be influencing stamen petaloid formation. To investigate the effect of DNA methylation on the stamen petaloid, NNU_05583 was identified in P vs St and P vs Sp, encoding an Auxin-induced protein X10A. This protein is a member of the small auxin up-regulated RNAs (SAUR) family and has a lower methylation level in the stamen petaloid than the stamen under DMR. In the lotus, the SAUR may control the variation of stamens and cell elongation. *SAUR* genes promoting cell expansion in *Arabidopsis* have been reported [37]. *SAUR63* regulates stamen filament elongation [38]. Most of the *SlSAUR* genes exhibit accumulating patterns of the transcript in the flower [39]. Thus, we predict that NNU_05583 could have specific involvement in influencing the stamen to convert to petal-like features.

**Figure 7:**
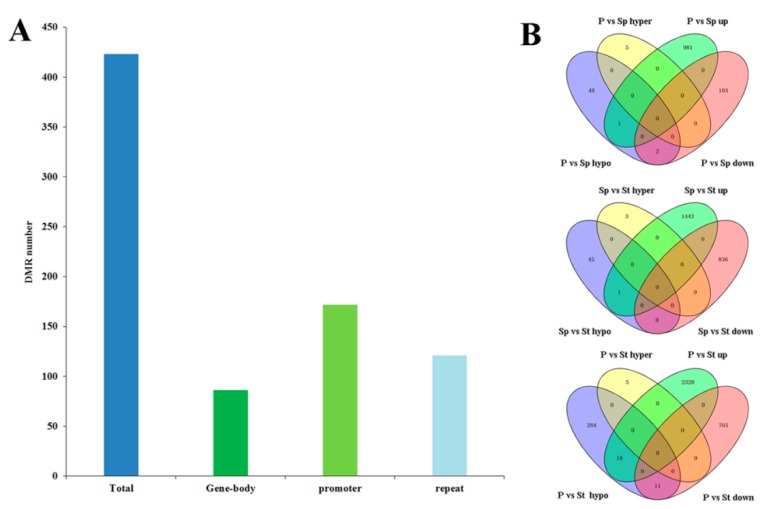
DNA methylation variation among organs. (**A**) The distribution of DMRs in the gene body, promoter (up or down 2 kb flanking regions) and repeat. (**B**) Venn diagram of DMR associated genes and DEGs in the comparisons of P vs Sp, Sp vs St and P vs St.

Collectively, we identified 415 DMRs (*p* < 0.05, Fisher’s exact test). About 20% of DMRs were located in the gene-body, including the exon and the intron, while 41% and 29% were located in the promoter and the repeat regions, respectively (Figure 7a). Regarding the relationship between DNA methylation and gene expression, we tested whether DMR could affect the expression of neighboring genes. The transcripts of all neighboring genes were confirmed in the three organs [21]. Based on the two-fold change criteria, a total of 982, 1444, and 2338 up-regulated DEGs, and 105, 836, and 776 down-regulated DEGs in comparisons of P vs Sp, Sp vs St, and P vs St, were identified, respectively (Figure 7b). A significant enrichment of up-regulated genes in the hypomethylated DMR-associated genes was observed in P vs Sp and Sp vs St. Only one gene was common in P vs Sp and Sp vs St, respectively (Figure 7b and Table S3). In P vs St, 313 hypomethylated DMR-associated genes were identified; eighteen of the up-regulated genes in the hypomethylated DMR-associated genes were significantly enriched (Figure 7b and Table S3). However, two and eleven down-regulated genes were hypomethylated in P vs Sp and P vs St, respectively, indicating that their differential accumulation was not affected by the methylation fluctuations. Recent *Arabidopsis* methylome analysis definitively revealed that DNA methylation only slightly affects gene expression [33]. A more recent study pointed out that differentially expressed genes are not associated with the corresponding methylation variations and suggested DNA methylation changes could directly and indirectly influence the gene expression in apples [34]. Comparison of the down-regulated or up-regulated genes with hypermethylated DMR-associated genes indicated the absence of down-regulated or up-regulated genes in the hypermethylated DMR-associated genes (*p* > 0.05, Fisher’s exact test) (Figure 7b). Hypomethylation could have a greater effect than hypermethylation in controlling gene expression in a lotus; this result is similar to previous findings for soybeans [13]. Since most of the differentially expressed genes had similar DNA methylation levels in different organs, we hypothesized that DNA methylation impacts on gene expression could be fundamentally regulated through specific transcription factors and other encoding proteins. This is consistent with the fact that most of the 31 candidate DMR-associated genes were annotated as protein-coding genes. Between P vs St and Sp vs St, one common DMR-associated down-regulated gene (NNU_03683) homolog of the receptor protein kinase ZmPK1 was identified (Figure 7b and Table S3). In *Arabidopsis*, the RPK2 encoding receptor-like protein kinase controls pollen development [40]. Another common DMR-associated up-regulated gene (NNU_05638), similar to the gene encoding U-box domain-containing protein 33, was identified (Figure 7b and Table S3). 

**Figure 8:**
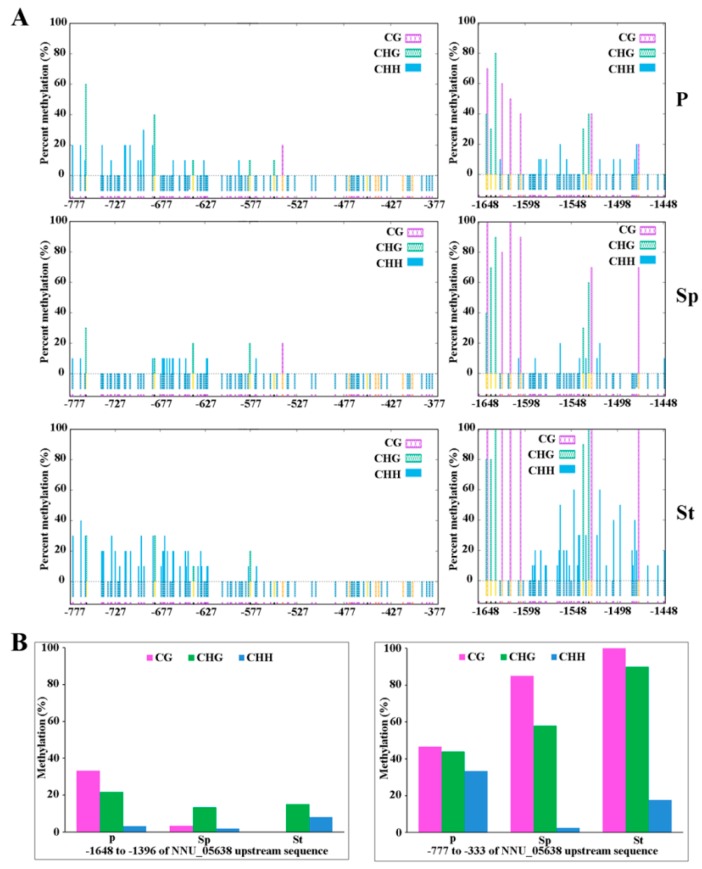
The methylation level of NNU_05638 promoter. (**A**) The data for the −777 to −377 and −1648 to −1448 regions in P, Sp, and St. (**B**) the percentage of methylation for the −777 to −377 and −1648 to −1448 regions in P, Sp, and St. Three different types of methylation site, i.e. CG, CHH and CHG, were analyzed.

The Plant U-box (PUB) domain-containing protein is involved in protein ubiquitination, which is linked with almost every cellular process [41]. The single mutant *pub4* and double mutant *pub2 pub4* exhibited a reduced number of stamens and incomplete tapetum cells [42,43]. *PUB4* contributes to the maintenance of shoot apical meristem (SAM) development [44]. This result indicates that *PUB*s may contribute to stamen and SAM development in the lotus. The promoter of NNU_05638 DNA methylation shows hypomethylation in Sp vs St (Figure S6a). Correspondingly, NNU_05638 was upregulated in Sp vs St (Figure S6b), implying that NNU_05638 influences the cellular process in floral organ development, promoting stamen to be petaloid through changes in DNA methylation. The phenomenon is consistent with the previous study, which reported that plants with reduced methylation result in floral organs abnormalities, like staminoid petals [45]. To further verify the differences in the DNA methylation of NNU_05638 in floral organs, we analyzed the methylation levels of the NNU_05638 promoter region by traditional bisulfite sequencing. We found that the methylation profile in Sp was significantly different compared to St (Figure 8a,b). This is consistent with the result of WGBS, suggesting that the data of WGBS is reliable. Furthermore, two regions (−777 to −333 and −1648 to −1396) exhibited distinct methylation statuses, including methylation levels and sites among three floral organs (Figure 8). Specifically, the methylation levels in the −1648 to −1396 region were distinctly changed in P, Sp, and St. Simultaneously, the efficiency of methylation in Sp was reduced compared with that of St. However, NNU_05638 functional analysis needs to be carried out in future.

## 3. Conclusions

In summary, the DNA methylation profiles in the petal, stamen petaloid, and stamen of a lotus provided clues for further understanding the genetic pathway regulatory roles in floral organ formation. Our results indicate that the stamen petaloid is derived from the stamen and similar to a petal. The profile of DNA methylation in these three lotus organs suggests that the effects of DNA methylation were located in the stamen petaloid. The methylation level of different floral organs exhibited significant changes in the CHH context. In addition, the methylation level in introns was higher compared to that in exons. Under DNA methylation association with RNA-Seq data, 31 DMR-associated genes were identified. In particular, a U-box domain-containing protein 33 encoding gene might play crucial roles in floral organ formation. Future functional studies are required to elucidate the process of DNA methylation and gene expression. We propose conduction of ChIP-seq for the candidate genes to elucidate the potential interaction with other regulatory factors involved in DNA methylation and demethylation reactions. Additionally, application of a DNA methylation inhibitor in the flower bud of lotus before the stamen differentiation phase could be carried out, to check if this causes an increase of the stamen petaloid, which will aid in obtaining further insight into the significance of DNA methylation for the conversion of stamens to petaloid-like organs.

## 4. Materials and Methods

### 4.1. Plant Materials

Petals, stamen petaloids, and stamens of the sacred lotus ‘Fenhonglingxiao’ (Figure 1) were collected at 10 AM in the Wuhan Botanical Garden, Chinese Academy of Sciences (WBGCAS), Hubei Province, China. The collected tissues were immediately frozen in liquid nitrogen and stored at −80 °C for further use. Genomic DNA was extracted from a lotus using the cetyltrimethylammonium bromide (CTAB) method [46].

### 4.2. Whole-Genome Bisulfite Sequencing

Bisulfite sequencing was performed to identify genome-wide methylation. 5.2 μg of purified genomic DNA was fragmented by Covaris S220, followed by the addition of dA to the 3’-end. According to the manufacturer’s instructions, cytosine-methylated adapters were ligated to the sonicated DNA. The ligation products were treated twice with bisulfite using an EZ DNA Methylation-Gold^TM^ Kit (Zymo Research). The single-strand DNA fragments were PCR amplified using KAPA HiFi HotStart Uracil+ReadyMix (2×). The resultant DNAs were quantified using qPCR (Life Technologies, CA, USA), and the insert size was assayed on the Agilent Bioanalyzer 2100 system. Bisulfite sequencing (BSeq) libraries were performed on a cBot Cluster Generation System using TruSeq PE Cluster Kit v3-cBot-HS (Illumia) according to the manufacturer’s instructions. The whole-genome bisulfite sequencing of the three samples and mapping and the processing of the reads was performed by Beijing Novogene Bioinformatics Technology Co., Ltd. Using Illumina Hiseq 2000/2500; the libraries were constructed after cluster generation and 125 bp paired-end reads were generated. Image analysis and base calling were performed with the Illumina CASAVA pipeline, and finally 125 bp paired-end reads were generated. Sequencing data was deposited in the National Center for Biotechnology Information (NCBI) under the accession number PRJNA453917.

### 4.3. Bioinformatic Analysis of Whole-Genome Bisulfite Sequencing Data

The remaining reads with remove adapters, Ns and low-quality reads were counted as clean reads and stored in the FASTQ format. The Bismark software (version 0.16.1) was used to map bisulfite-treated reads to the reference genome of the lotus with the default parameters [22,23].

To identify the methylation site, we modeled the sum s^+^_i,j_ of methylated counts as a binomial (Bin) random variable with methylation rate r_i,j_, s^+^_i,j_ ~ Bin (s^+^_i,j_ + s^-^_i,j_, r_i,j_). To calculate the methylation level of the sequence, we divided the sequence into multiple bins, with bin size being 10 kb. The sum of the methylated and unmethylated read counts in each window was calculated. The methylation level (ML) for each window or C site shows the fraction of methylated Cs, and is defined as: ML (C) = reads (mC) / reads (mC + umC).

Differentially methylated regions (DMRs) among P, Sp, and St were identified using the swDMR software (http://122.228.158.106/swDMR/), which uses a sliding-window approach. The window is set to 1000 bp and the step length is 100 bp. A Fisher test was implemented to detect the DMRs (*p* value < 0.01). Gene Ontology (GO) enrichment analysis of genes related to DMRs was implemented by the GOseq R package [47], in which gene length bias was corrected. GO terms with a corrected *p*-value less than 0.05 were considered significantly enriched by DMR related genes.

### 4.4. Differential Expression Analysis of mRNA Sequencing Data and Confirmation by qRT-PCR

The analysis of the RNA-seq data of P, Sp, and St was based on clean data of a high quality. The transcriptome sequencing data were deposited in PRJNA417869. Differential expression analysis of the two samples was performed and approached for controlling a fold change of no less than two and the false discovery rate (FDR ≤ 0.05). Any genes with an adjusted *p*-value < 0.05 were assigned as differentially expressed. The qRT-PCR reactions were performed on the Bio-Rad using SYBR (BioRad, http://www.bio-rad.com/). The reaction was initiated at 95 °C for 10 s, followed by 40 cycles of 95 °C for 15 s, 60 °C for 15 s and 72 °C for 30 s. Fluorescent products were detected in the last step of each cycle. Melting curve analysis was performed at the end of 40 cycles to ensure proper amplification of target fragments. The data presented for qRT-PCR experiments are the average relative quantities from three biological replicates, where every biological replicate is the mean of three technical repeats. Relative gene expressions were normalized by comparison with the expression of lotus β-actin (NNU-24864) and analyzed using the 2^-ΔΔCT^ method. The data was indicated as mean ±SD. The primers were listed in Table S4.

### 4.5. Validation of the WGBS Result about Methylation of the NNU_05638 Promoter

To verify the WGBS result of NNU_05638, the traditional bisulfite sequencing method was used. The methylation of the NNU_05638 promoter was analyzed in three different tissues (Petal, Stamen petaloid, and Stamen) according to the method described by Deng et al. [17]. Briefly, less than 500 ng of purified genomic DNA was mixed with sodium bisulfite to modify the substance and then purified by an EpiTect® Bisulfite kit (Qiagen) following the protocol. Subsequently, the treated DNA as a template was prepared for PCR. A reoughly 2 kb region of the NNU_05638 promoter was amplified using three primer pairs (Table S5), which were designed by Methyl Primer Express v1.0. The total volume for the PCR was 25 μL. Using rTaq (TaKaRa), the PCR programs were carried out as follows: 94 °C, 5 min followed by 40 cycles of 94 °C, 30 s, 58 °C, 30 s, and 72 °C, 30 s, and then 72 °C, 10 min. Then, the PCR products were ligated into the pMD18-T vector after 16 °C incubating overnight and transformed into DH5α. 10 positive clones of each sample were used for sequencing. The percentage methylation of cytosine (C) in all cytosine contexts was analyzed by the online software Bisulfite Analysis (http://katahdin.mssm.edu/kismeth/revpage.pl). The fraction methylated C was calculated by 100 × C / (C + T) (C means methylated C, and T was converted by unmethylated C).

## Supplementary Materials

The following are available online at https://www.mdpi.com/2223-7747/8/5/135/s1, Figure S1: The proportion of mCGs, mCHGs, and mCHHs in plants, Figure S2: Sequence preferences for methylation in CG, CHG, and CHH contexts, Figure S3: The distribution of methylated cytosine in all contexts in the main chromosome, Figure S4: A circos plot of DNA methylation in P vs Sp, Sp vs St, and P vs St, Figure S5: The DNA methylation patterns in gene regions in DEGs, Figure S6: IGV software depicts the demethylation of NNU_05638 promoter region promoting stamen to petaloid (A) and the methylation difference and expression analysis of NNU_05638 in P, Sp, and St (B), Table S1: Summary of sequencing and reads, Table S2: Summary of methylcytosines in each sequence context, Table S3: List of hypomethylated DMR-associated genes in all organs, Table S4: The primers for qRT-PCR, Table S5: Three primers designed for bisulphate sequencing PCR on the promoter of NNU_05638.

## Abbreviations

BSeq: Bisulfite sequencing;
CMT: CHROMOMETHYLASE;
CTAB: cetyltrimethylammonium bromide;
DEGs: differential expression genes;
DMR: differential methylation region;
DNMT1: DNA methyltransferase 1;
DRM: DOMAINS REARRANGED METHYLTRANSFERASE;
FPKM: fragments per kilo base of transcript per million base pairs sequenced;
GO: gene ontology;
H3K9: histone H3 lysine 9;
KEGG: kyoto encyclopedia of genes and genomes;
mCs: methylcytosines;
MET1: methyltransferase 1;
P: petal;
PUB33: Plant U-box 33;
qRT-PCR: quantitative real time PCR;
RdDM: RNA-directed DNA methylation;
SAM: shoot apical meristem;
SAUR: small auxin up-regulated RNAs;
siRNAs: short-interfering RNAs;
Sp: petaloid stamen;
St: stamen;
TSSs: transcription starting sites;
WGBS: whole genome bisulfite sequencing.
