# Supplementary material for: Genome-Wide DNA Methylation Profiling in the Lotus (Nelumbo nucifera) Flower Showing its Contribution to the Stamen Petaloid
Source: Plants (Basel). 2019 May 20;8(5):135. doi: 10.3390/plants8050135 (PMC6572404; doi:10.3390/plants8050135)
Supplement: Supplementary file 1 [file plants-08-00135-s001.pdf]

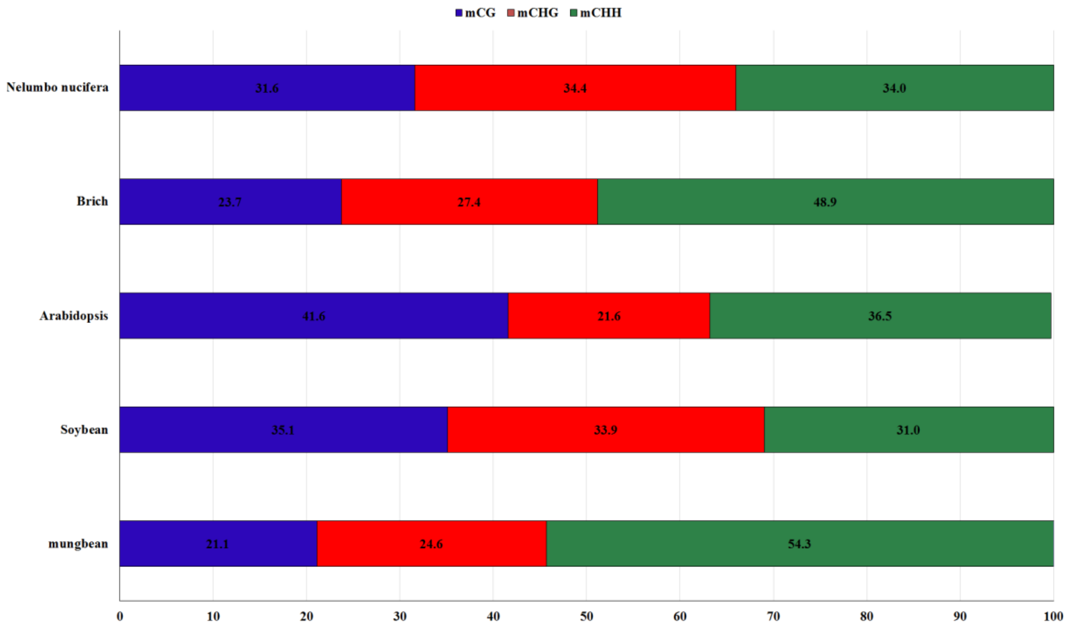

Figure S1. The proportion of mCGs, mCHGs, and mCHHs in plants.

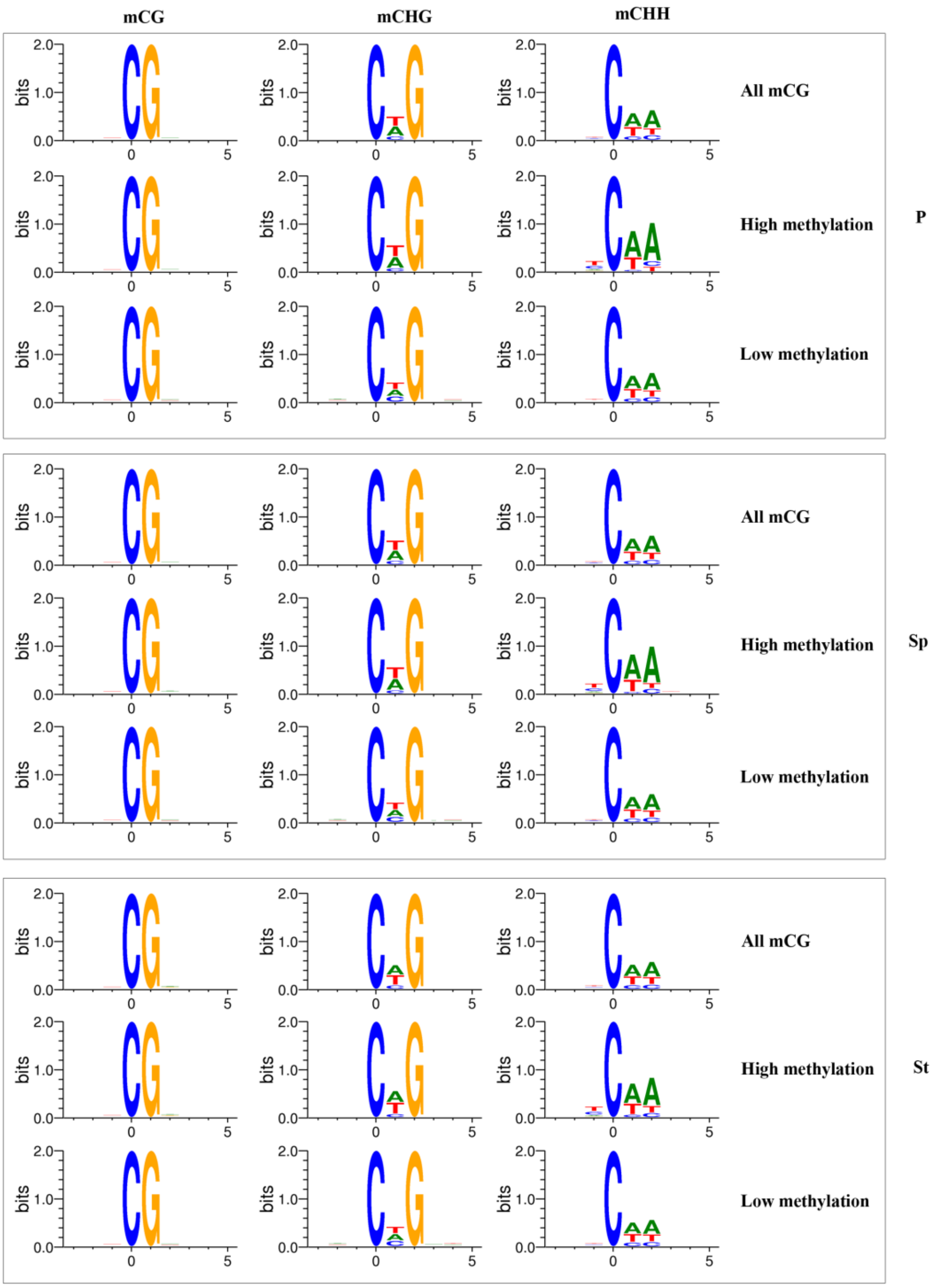

Figure S2. Sequence preferences for methylation in CG, CHG, and CHH contexts. The mC site methylation level was divided into three groups: All mC sites; High Methylation (ML ≥ 0.75), Low Methylation (ML < 0.75). ML indicated methylation level.

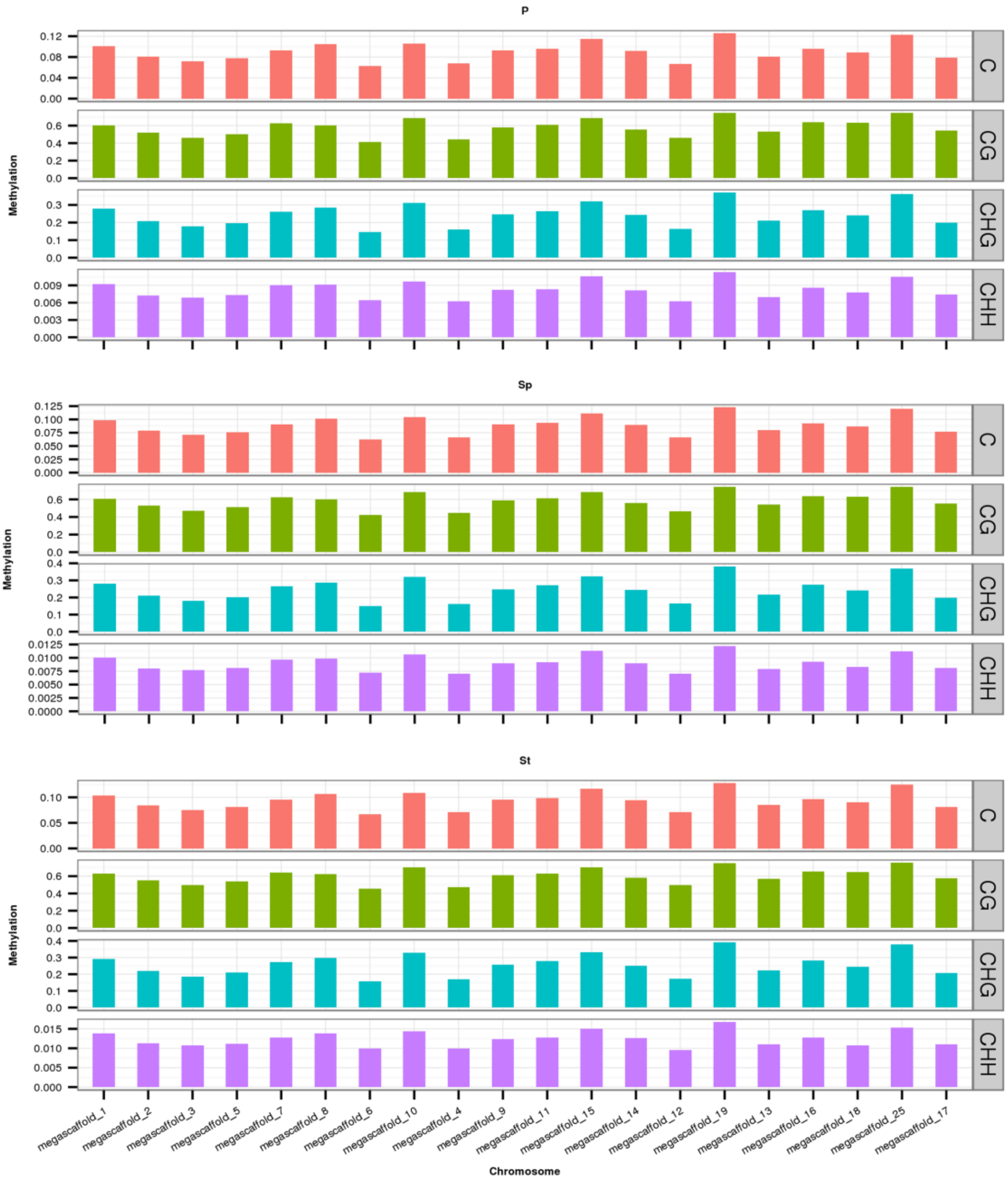

Figure S3. The distribution of methylated cytosine in all contexts in the main chromosome.

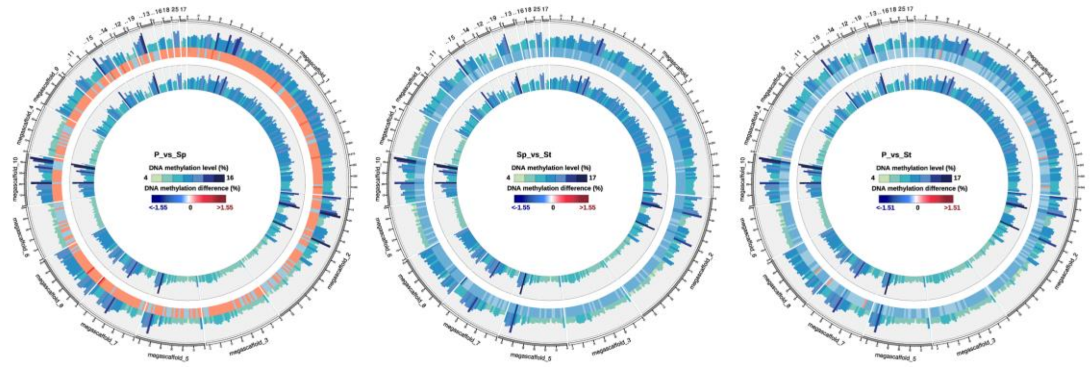

Figure S4. A circos plot of DNA methylation in P vs Sp, Sp vs St, and P vs St.

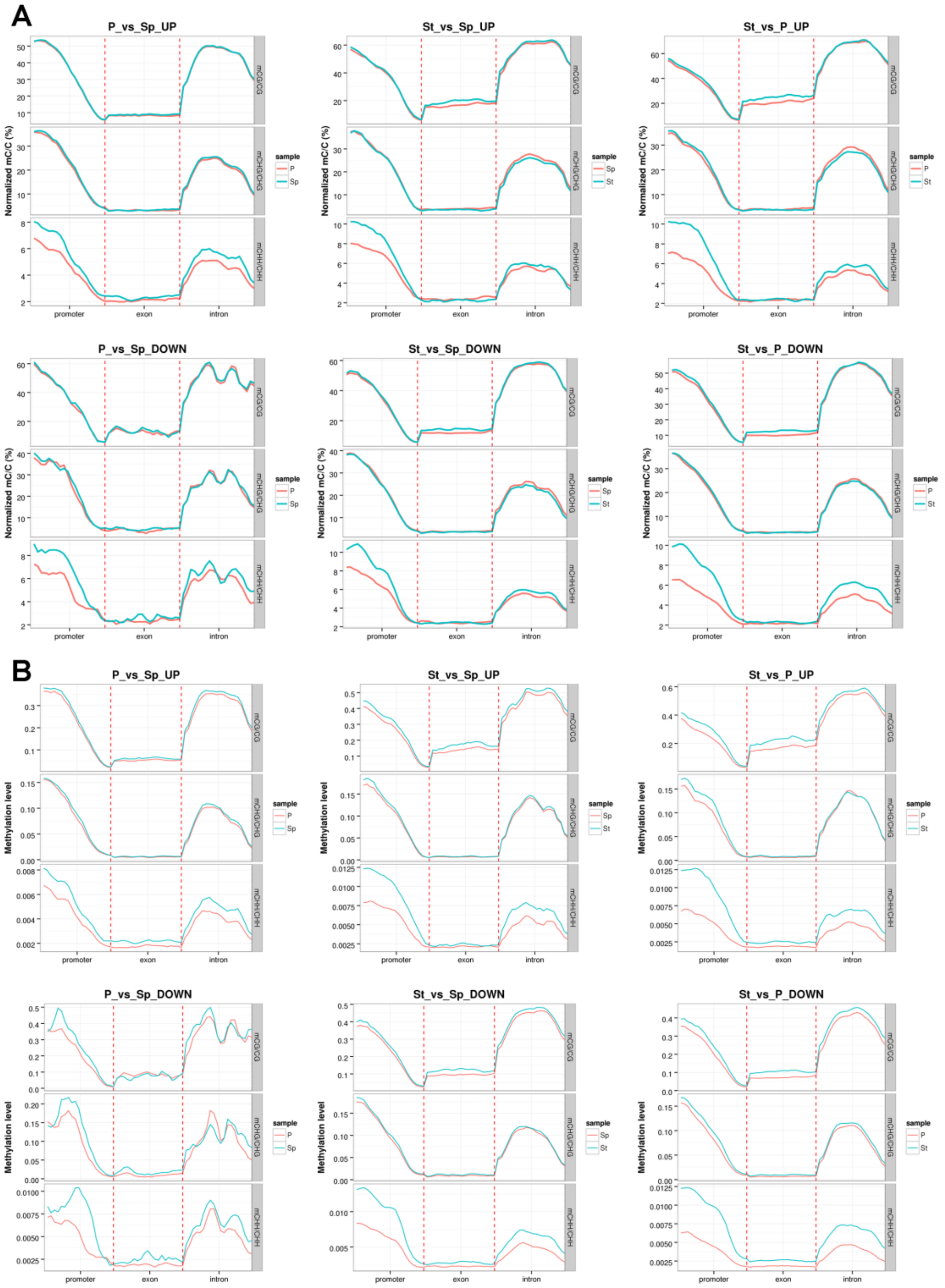

Figure S5. The DNA methylation patterns in gene regions in DEGs. (A) The DNA Methylation density of DEGs (P vs Sp, St vs Sp, and St vs P) throughout the gene regions in different floral organs. **(B)** The DNA Methylation level of DEGs (P vs Sp, St vs Sp, and St vs P) throughout the gene regions in different floral organs.

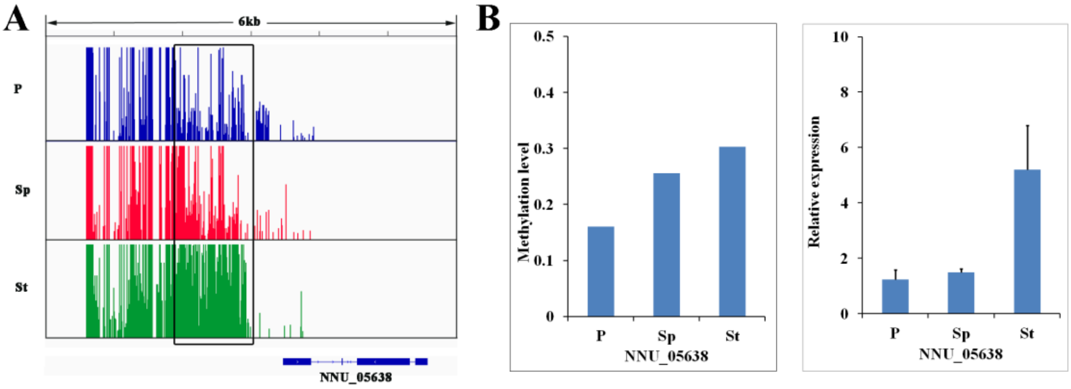

Figure S6. IGV software depicts the demethylation of NNU_05638 promoter region promoting stamen to petaloid (A) and the methylation difference and expression analysis of NNU_05638 in P, Sp, and St (B).

Table S1. Summary of sequencing and reads.

Sample name	Raw Reads	Raw Bases (G)	Clean Reads	Clean Bases (G)	Clean ratio (%)	Q20 (%)	GC Content (%)	BS Conversion Rate (%)	Mapped reads	Mapping rate (%)	Duplication rate (%)	Genome coverage mean	Cytosines coverage mean
P	122171421	36.65	116929861	32.55	88.81	96.1	22.23	99.835	88738071	75.89	8.89	21.26	12.7
Sp	119687017	35.91	115231027	32.24	89.78	96.34	21.54	99.809	86089100	74.71	9.00	20.68	12.3
St	117771103	35.33	113981900	31.84	90.12	96.45	21.37	99.778	86797216	76.15	9.33	20.74	11.9

Table S2. Summary of methylcytosines in each sequence context.

Sample	P		Sp		St		Mean
	proportion of mCs	reads	proportion of mCs	reads	proportion of mCs	reads	proportion of mCs
**mCG**	33.14%	15307291	31.66%	14635358	30.04%	13602715	31.61%
**mCHG**	35.83%	16554080	34.94%	16150150	32.42%	14681366	34.40%
**mCHH**	31.03%	14334879	33.39%	15434846	37.54%	16999686	33.99%

Table S3. List of hypomethylated DMR-associated genes in all organs.

Hypomethylated DMR-associated up-regulated genes in P vs Sp

Gene ID	Annotation
**NNU_14779**	uncharacterized protein LOC104603194 [Nelumbo nucifera]

Hypomethylated DMR-associated down-regulated genes in P vs Sp

Gene ID	Annotation
**NNU_03683**	PK1: Putative receptor protein kinase ZmPK1 (Zea mays)
**NNU_25355**	ARA12: Subtilisin-like protease (Arabidopsis thaliana)

Hypomethylated DMR-associated up-regulated genes in Sp vs St

Gene ID	Annotation

**NNU_05638**	PUB33: U-box domain-containing protein 33 (Arabidopsis thaliana)

Hypomethylated DMR-associated up-regulated genes in P vs St

Gene ID	Annotation
**NNU_00424**	Protein of unknown function
**NNU_00560**	gluP: Rhomboid protease gluP (Bacillus subtilis)
**NNU_01525**	Protein of unknown function
**NNU_01772**	CYP71A1: Cytochrome P450 71A1 (Persea americana)
**NNU_03180**	V-type proton ATPase catalytic subunit A (Daucus carota)
**NNU_03373**	GSVIVT00027310001: UPF0497 membrane protein 16 (Vitis vinifera)
**NNU_05638**	PUB33: U-box domain-containing protein 33 (Arabidopsis thaliana)
**NNU_09335**	AGD5: Probable ADP-ribosylation factor GTPase-activating protein AGD5 (Arabidopsis thaliana)
**NNU_11143**	LAL5: MATE efflux family protein LAL5 (Arabidopsis thaliana)
**NNU_14184**	AGD11: Probable ADP-ribosylation factor GTPase-activating protein AGD11 (Arabidopsis thaliana)
**NNU_14834**	Putative clathrin assembly protein At1g25240 (Arabidopsis thaliana)
**NNU_15184**	Protein of unknown function
**NNU_16725**	ADH: Alcohol dehydrogenase (Fragaria ananassa)
**NNU_19124**	DREB1E: Dehydration-responsive element-binding protein 1E (Arabidopsis thaliana)
**NNU_21147**	ELI3: Probable mannitol dehydrogenase (Mesembryanthemum crystallinum
**NNU_22678**	ZNF828: Zinc finger protein 828 (Homo sapiens)
**NNU_23350**	PXL2: Leucine-rich repeat receptor-like protein kinase PXL2 (Arabidopsis thaliana)
**NNU_24014**	SRPK1: Serine/threonine-protein kinase SRPK1 (Homo sapiens)

Hypomethylated DMR-associated down-regulated genes in P vs St

Gene ID	Annotation
**NNU_08735**	SEC22: 25.3 kDa vesicle transport protein (Arabidopsis thaliana)
**NNU_20806**	Protein of unknown function
**NNU_19557**	Probable peptide/nitrate transporter At1g72125
**NNU_13621**	Uncharacterized protein At4g06744 (Arabidopsis thaliana)
**NNU_01487**	4-coumarate--CoA ligase 2 (Glycine max)
**NNU_08358**	SCPL35: Serine carboxypeptidase-like 35 (Arabidopsis thaliana)
**NNU_03683**	PK1: Putative receptor protein kinase ZmPK1 (Zea mays)
**NNU_07659**	PSRP3: 30S ribosomal protein 3%2C chloroplastic (Spinacia oleracea)
**NNU_00792**	bysl: Bystin (Nematostella vectensis)
**NNU_15841**	GGR: Geranylgeranyl pyrophosphate synthase-related protein%2C chloroplastic (Arabidopsis thaliana)
**NNU_08534**	UBC4: Ubiquitin-conjugating enzyme E2 4 (Arabidopsis thaliana)

Table S4. The primers for qRT-PCR.

Gene name	Forward primer (5′-3′)	Reverse primer (5′-3′)
**NNU_01774**	GATGGTGCTGAGGGTGAGTG	ATCAACAGCCCACCTCGTAA
**NNU_11434**	TGATGTTGTGACGGTAGCCC	CGGTAAACCCTCCAGAAGAC
**NNU_17480**	CTATGGTCGCTTGTTTCCTG	TCTGAGGCACTTGCGTAATAG
**NNU_17715**	GATTATCCCACCGCTCACTC	AAAGCCTCGCACCAGTCG
**NNU_21025**	ATGCTATGAGGATGGGACA	ATTCATTGCTGTTTGCCAC
**NNU_24864**	ATACGCCAGTGGTAGAACAAC	CAGCAAGGTCCAACCGAAG
**NNU_05638**	TGAAAGGATGGCTGGAGGG	AACGAAGAGCGTGATTAGGGA

Table S5. Three primers designed for bisulphate sequencing PCR on the promoter of NNU_05638.

Primer site (bp)	Forward primer (5′-3′)	Reverse primer (5′-3′)
**-1648 to -1396**	GGGATTAAATTTGGATTGAAGTTTA	AACCACCCACCTTTTTAAAATAAA
**-777 to -333**	TTATTTTGGTTATTGGTGTAAATGT	CTACTAAACCCATTAAATCCAATTT
**-348 to -61**	TTAATGGGTTTAGTAGTGGAGG	ATTCRATAACAAAATCTATAAACAAA
